# The Challenges of Establishing an Optimal Link Between Nutritional Requirements, Dietary Knowledge and Culinary Skills in Chronic Kidney Disease: An Exploratory Review of the Literature

**DOI:** 10.3390/nu18142245

**Published:** 2026-07-09

**Authors:** Jorge Casaña Mohedo, Elena Sandri

**Affiliations:** 1 Faculty of Medicine and Health Sciences, Catholic University of Valencia San Vicente Mártir, c/Quevedo, 2, 46001 Valencia, Spain; jorge.casana@ucv.es; 2 SONEV Research Group, Faculty of Medicine and Health Sciences, Catholic University of Valencia San Vicente Mártir, C/Quevedo, 2, 46001 Valencia, Spain; 3 Department Nursing, Catholic University of Valencia San Vicente Mártir, c/Quevedo, 2, 46001 Valencia, Spain; 4 Italian Society of Nephrology Nurse (SIAN), Via Capotesta 1/30, 07026 Olbia, Italy

**Keywords:** chronic kidney disease, nutritional requirements, dietary knowledge, food literacy, culinary skills, culinary medicine, renal diet, hemodialysis, peritoneal dialysis, dietary adherence, scoping review

## Abstract

**Background/Objectives:** Chronic kidney disease (CKD) requires complex Medical Nutrition Therapy, yet dietary non-adherence remains high (20–70%) due to a gap between theoretical knowledge and practical application. This scoping review aims to map the literature exploring the intersection between nutritional requirements, dietary knowledge (food literacy), and culinary skills in adults with CKD (stages 3–5 and dialysis). **Methods:** Following JBI methodology and PRISMA-ScR guidelines, a systematic search was conducted across PubMed, Web of Science, Scopus, and EBSCO. Studies were screened using the PCC (Population, Concept, Context) framework and mapped according to the WHO International Classification of Functioning, Disability and Health (ICF). **Results:** Out of 617 identified records, 49 studies from 24 countries were included. Findings reveal that while high-income regions focus on e-Health and precision technology, developing regions prioritize visual literacy and culturally adapted analog tools. Culinary skills, such as leaching and moist-heat cooking, represent practical strategies for reducing mineral bioavailability; however, the evidence directly linking these skills to clinical outcomes remains fragmented. However, culinary skills remain the least represented component in the current literature, despite being a critical facilitator for adherence. **Conclusions:** Current literature has not yet established a robust empirical link between food literacy and culinary skills in CKD. This review maps a precise research agenda: future large-scale, validated studies must characterise this relationship before scalable culinary medicine interventions can be designed and evaluated.

## 1. Introduction

Chronic kidney disease (CKD) is a global public health crisis, currently affecting an estimated 850 million people worldwide [1]. The condition is associated with severe metabolic disturbances, chronic comorbidities, and a significantly increased risk of cardiovascular morbidity and mortality [2,3]. In both conservative management without dialysis and across all modalities of renal replacement therapy, Medical Nutrition Therapy (MNT) is recognized as a foundational pillar of care [4,5]. Adequate nutritional management is essential to prevent protein-energy wasting (PEW), delay the progression of renal decline, and mitigate uremic symptoms [6,7].

Traditional dietary guidelines for CKD have strictly focused on the rigorous monitoring and restriction of specific nutrients, primarily sodium, potassium, phosphorus, fluid, and protein [8,9]. Consequently, the renal diet is widely recognized by health professionals and patients alike as one of the most complex, restrictive, and emotionally demanding therapeutic regimens in chronic disease management [10,11]. This overwhelming complexity often leads to alarmingly low rates of dietary adherence. Current literature estimates that dietary non-adherence among patients with CKD ranges widely between 20% and 70%, with the weighted mean adherence to end-stage kidney disease (ESKD) dietary recommendations reported as low as 31.5% [12,13]. Dietary non-adherence is a multidimensional phenomenon driven by a complex interplay of patient-related, condition-related, socio-economic, and healthcare system factors [1,14]. While patients receive extensive educational materials outlining dietary restrictions, a profound gap exists between theoretical nutritional knowledge and practical application [15,16].

Studies consistently indicate that mere awareness of dietary principles does not effectively translate into behavior change [17,18]. Patients frequently encounter significant barriers to adherence, including taste aversions to modified or boiled foods, lack of familial and social support, and financial constraints that limit access to recommended fresh dietary items [14,19]. Furthermore, conventional renal diets often severely compromise the sensory quality and palatability of meals. The strict restriction of sodium and spices, combined with the necessity of utilizing aggressive thermal processing techniques such as prolonged soaking or double-boiling to reduce mineral loads, strips meals of their natural flavors and textures. This profound loss of food enjoyment inevitably leads to ‘dietary fatigue,’ an emotional and sensory exhaustion that drives patients toward therapy abandonment and an increased, dangerous reliance on highly palatable but toxic ultra-processed foods (UPFs) [20,21]. These UPFs are highly dangerous in the CKD population, as they are typically loaded with highly bioavailable, hidden phosphorus and potassium additives, and are strongly associated with higher mortality and progression of the disease [4,22].

To bridge the gap between theoretical dietary knowledge and actual food consumption, the concept of “food literacy” has gained critical importance. Food literacy encompasses the functional, interactive, and critical knowledge, skills, and behaviors required to plan, select, prepare, and consume a high-quality diet [11,23]. Patients and their caregivers who possess higher food literacy demonstrate better adherence to nutritional guidelines, make healthier food choices, and experience a lower burden when managing the multifaceted dietary needs of CKD [24,25]. However, a substantial proportion of patients with CKD exhibit limited food and nutritional literacy (up to 46.3% in some hemodialysis cohorts), pointing to an urgent need for interventions that go beyond theoretical education [26].

In response to these ongoing challenges, “culinary medicine” has emerged as a novel, evidence-based discipline that blends the traditional art of cooking with the science of nutrition and medicine [27,28]. The goal of culinary medicine in nephrology is to empower patients and caregivers with practical skills to prepare palatable, kidney-protective meals at home, shifting the focus from isolated nutrient restriction to holistic meal preparation [6,27]. This shift is particularly relevant given the recent updates in CKD nutrition guidelines, such as the 2020 National Kidney Foundation Kidney Disease Outcomes Quality Initiative (KDOQI) [4]. These guidelines advocate for a more personalized approach that embraces plant-dominant low-protein diets (PLADO) and encourages the consumption of fruits and vegetables to reduce metabolic acidosis, lower the acid load, and positively modulate the gut microbiome [6,16].

Transitioning to these plant-based dietary patterns while managing potassium and phosphorus safely demands specific culinary techniques. For example, culinary strategies such as leaching vegetables to extract potassium, or substituting salt with aromatic herbs and spices (e.g., garlic or onion powder) to compensate for sodium restriction without sacrificing flavor, are essential skills [1,25,29]. Despite the acknowledged benefits of MNT and the emerging recognition of culinary medicine, the literature currently lacks a comprehensive synthesis of how theoretical dietary knowledge intersects with practical culinary skills to impact clinical outcomes in CKD. Healthcare providers, particularly nephrology nurses who spend hours with patients during dialysis treatments, are uniquely positioned to act as “culinary coaches” [30,31]. Yet, their roles in translating nutrition science into practical cooking skills and assessing food literacy remain underexplored [11,32].

Therefore, this scoping review aims to systematically map the existing literature to explore the intersection of these three critical domains. By highlighting the fundamental transition from “knowing what to eat” to “knowing how to cook it,” this review seeks to identify gaps in current research and inform future clinical strategies that integrate food literacy and culinary medicine into comprehensive, patient-centered nephrological care.

## 2. Materials and Methods

### 2.1. Study Design

A scoping review was conducted following the methodology proposed by the Joanna Briggs Institute (JBI) for scoping reviews, adapted from the original conceptual framework by Arksey and O’Malley (2005) [33] and subsequently developed by Levac et al. (2010) [34]. This design was selected for its suitability for mapping the scope, breadth, and nature of available evidence regarding an emerging or heterogeneous research field, identify knowledge gaps, and clarify key concepts, without restrictions based on the methodological design of the primary studies. The reporting of this review followed the recommendations of the PRISMA-ScR statement [35].

The research question was formulated in compliance with the PCC framework (Table 1) and was as follows: What is the extent of the evidence regarding the relationship between nutritional requirements, dietary knowledge, and culinary skills in individuals with Chronic Kidney Disease (stages 3–5 and dialysis) in clinical and community settings?

### 2.2. Protocol and Registration

The review protocol was developed a priori and registered in the Open Science Framework (OSF) under the identifier OSF zw3ut (https://osf.io/zw3ut/, accessed on 26 April 2026), as recommended to ensure the transparency of the process.

### 2.3. Eligibility and Population Selection Criteria

To ensure methodological homogeneity and clinical applicability of the synthesized findings, strict selection criteria were established to define the study population based on their pathophysiological profile and dietary self-management context.

- Inclusion Criteria: Studies were exclusively included if they were conducted on adult populations (≥18 years old) with a diagnosis of advanced CKD undergoing conservative or kidney-preserving management (Stages 3, 4, and 5 not on dialysis), as well as stable patients on chronic HD programs. Additionally, selected studies had to explicitly evaluate constructs related to food literacy, theoretical dietary knowledge, or practical culinary skills applied to managing macronutrient and electrolyte restrictions.
- Exclusion Criteria: In line with the need to prevent bias arising from markedly divergent quality of life dynamics and nutritional interventions, the following profiles were strictly excluded:

1. Kidney transplant recipients: Given that immunosuppressive therapy and the recovery of graft function substantially alter metabolic targets, thereby eliminating strict restrictions on the leaching of exogenous minerals.
2. Patients on isolated Peritoneal Dialysis (PD): Minor PD cohorts were excluded because behavioral barriers, nutrient loss through the peritoneal effluent, and flexibility in potassium and fluid intake differ profoundly from the life disruption and rigid restrictions experienced by patients on hemodialysis and low-protein conservative treatment.

### 2.4. Search Strategy and Information Sources

The identification of the literature was carried out through a systematic search in the PubMed/MEDLINE, Web of Science (WoS), Scopus, and EBSCO databases; no date restrictions were applied to the search. To ensure comprehensiveness, a Boolean search strategy was designed, combining MeSH (Medical Subject Headings) terms with free-text keywords, structured into three conceptual axes:

1. The underlying pathology: (“Chronic Kidney Disease” OR “Dialysis”).
2. The concept of interest: Integrated descriptors such as (“Food Literacy”, “Culinary Medicine”, “Cooking”, “Culinary skills”, “Food Agency”, “Kitchen Competencies”, “Food Preparation”, “Food Handling”, and “Dietary Knowledge”).
3. The setting concept was left unspecified to cover the maximum number of settings possible and to avoid being restricted by different nomenclatures across cultures or countries.

An example of the final syntax used in PubMed was: ((“Chronic Kidney Disease” OR “CKD” OR “Dialysis”) AND (“Food Literacy” OR “Culinary Medicine” OR “Cooking” OR “Culinary skills” OR “Food Agency” OR “Kitchen Competencies” OR “Food Preparation” OR “Food Handling” OR “Dietary Knowledge”)). Additionally, a manual search of the reference lists of the selected articles was performed (snowballing technique) to identify key studies that might have been omitted by the database algorithms.

### 2.5. Evidence Selection Procedure

The screening process was executed in three successive stages to minimize selection bias. In the pre-selection phase, results were consolidated in a bibliographic manager, and duplicates were automatically removed. Subsequently, two reviewers independently assessed the suitability of the studies by reading the titles and abstracts, applying strict exclusion criteria to discard pharmacological clinical trials, single case studies, or narrative reviews that did not provide predictive empirical data. In the eligibility phase, the full text of the pre-selected articles was analyzed to verify that they met the requirement of specifically reporting nutritional and culinary models. The final consensus on inclusion was reached through technical discussion, ensuring that the final sample provided adequate representativeness of CKD.

### 2.6. Evidence Analysis and Synthesis

Given the nature of the included studies, the synthesis of results was conducted through a qualitative thematic analysis supported by quantitative evidence. The findings were categorized into three critical axes: nutritional requirements, dietary knowledge/literacy, or culinary skills/practical competencies within the context of CKD; and lastly, studies conducted in clinical or community settings, without geographical restriction.

### 2.7. Risk of Bias Assessment and Methodological Quality

Although scoping reviews do not always mandate an assessment of the quality of evidence (unlike systematic reviews), this study opted to conduct one to strengthen the validity of the conclusions. The Joanna Briggs Institute (JBI) Critical Appraisal Tool for analytical and cross-sectional studies was utilized [36]. Included studies were classified according to their risk of bias (Low, Moderate, or High). Those with a high risk in sample description or instrument validation were analyzed with caution during the synthesis of results.

The systematic search across the four electronic databases yielded an initial total of 617 records. The distribution of preliminary findings was: Scopus (*n* = 318), PubMed (*n* = 246), EBSCO (*n* = 47), and Web of Science (*n* = 6). Following the initial screening of titles and abstracts, 65 potentially relevant reports were identified. After an exhaustive full-text evaluation to ensure compliance with pre-established inclusion criteria, 47 studies were successfully included. Additionally, a manual search using the ‘snowballing’ technique was carried out, identifying 2 additional studies that met the eligibility criteria.

Regarding the database-specific yield reported in Table 2, the final inclusion rate of zero articles from the Scopus database warrants methodological clarification. Scopus represents an expansive multidisciplinary index with a high degree of content intersection and overlapping coverage with specialized databases such as PubMed/MEDLINE and Web of Science. During the initial screening and automated deduplication process, a massive volume of redundant records was identified. To preserve structural consensus and maintain unambiguous cataloging, priority for full-text eligibility was systematically allocated to the primary biomedical source (PubMed/MEDLINE). Consequently, while Scopus successfully identified a substantial portion of the relevant literature during the initial identification stage (*n* = 318), 100% of its eligible records were found to be duplicates of already selected articles, mathematically resulting in zero unique additions from this source in the final synthesis stage.

## 3. Results

### 3.1. Study Selection Procedure

The selection process was conducted in a systematic and transparent manner to ensure replicability. In the initial phase, duplicate records were removed using bibliographic management software (Zotero, version 9.0.5). Subsequently, two independent reviewers performed a screening of titles and abstracts based on the predefined inclusion criteria using the Rayyan web app (https://www.rayyan.ai). Articles that raised doubts or met the criteria proceeded to the full-text review phase. During this stage, the suitability of the studies was evaluated, specifically considering the use of structural equation modeling or path analysis. Any discrepancies between reviewers during the selection process were resolved through the intervention of a third reviewer or by consensus following a technical discussion. The studies finally included, along with all their characteristics, can be found in the Supplementary Materials.

**Figure 1:**
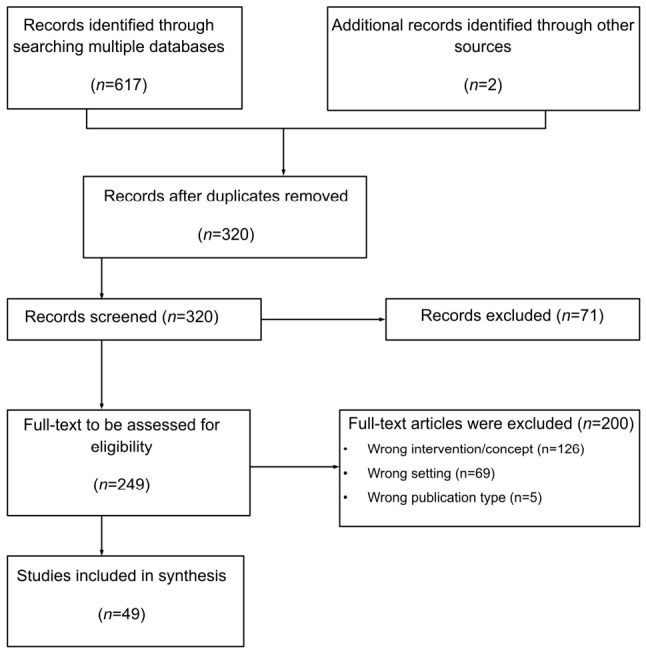
PRISMA Flow chart.

The study selection process is presented in the flow diagram [36] following the PRISMA-ScR criteria (Figure 1).

### 3.2. Geographical Distribution and Patient Demographics

Following a strict epidemiological consolidation to minimize sample heterogeneity, the final synthesis maps a consolidated clinical cohort of 2410 adult patients across 24 countries. Within this consolidated cohort of 2410 patients, nutritional targets varied significantly depending on the clinical stage. We estimate that 16.6% (*n* = 400) of the total mapped population—exclusively those under conservative management (CKD stages 3–5)—were actively prescribed a Low Protein Diet (LPD) or Very Low Protein Diet (VLPD). The remainder of the cohort predominantly comprised patients on maintenance hemodialysis, whose nutritional management prioritized mineral and fluid restrictions rather than strict protein limitation due to dialytic losses. Rather than evaluating research impact solely by the volume of published papers, analyzing the aggregated sample size (*N*) by geographical region highlights a profound shift in global renal nutrition research paradigms.

The vast majority of the synthesized population is concentrated in East and Southeast Asia (*N* = 1155 patients) and the Middle East (*N* = 812 patients). In these regions, research is overwhelmingly characterized by practical, clinical experimentation executed directly within dialysis centers. These studies focus heavily on testing interactive behavioral methods—such as the teach-back pedagogical method, social-media-delivered mHealth platforms, and video-assisted training—to optimize fluid and mineral self-management during treatment hours.

**Figure 2:**
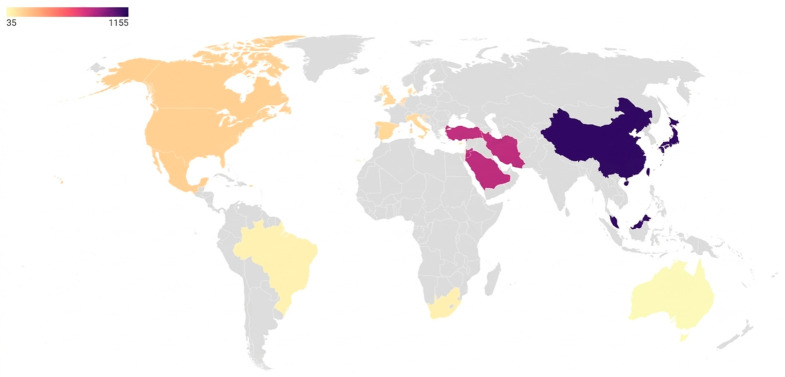
Global choropleth map highlighting the regional epidemiological distribution and aggregated sample size (*N* = 2410) of adult patients with advanced chronic kidney disease and maintenance hemodialysis across the synthesized literature.

Conversely, Western high-income regions exhibit a different focus despite smaller clinical trial sample sizes. Literature from North America (*N* = 186 patients) and Europe (*N* = 159 patients) shifts toward establishing situational diagnoses of socioeconomic inequalities, food insecurity, and low health literacy barriers. These regions focus on technological precision, evaluating tools like web-based self-monitoring portals and digital health applications, while rigorously assessing the bromatological efficacy of moist cooking methods to clear phosphorus and potassium additives. Lastly, in the Global South (*N* = 63 patients in South America and Africa), resource constraints drive a focus on cultural adaptation and visual literacy, prioritizing analog, text-free educational tools over high-technology platforms. This global distribution underscores that while the necessity of integrating culinary skills and dietary knowledge is universal, implementation models must be calibrated to the economic realities and healthcare systems of each specific region (Figure 2).

To provide a rigorous epidemiological perspective and fulfill recent methodological recommendations, the geographical analysis was shifted from a simple study-count distribution to an aggregation of the actual clinical sample size (N) across the distinct regions. This approach minimizes data dispersion and highlights the true population impact of the synthesized evidence. Consequently, Table 3 systematically outlines the distribution of the consolidated cohort of 2410 adult patients with advanced CKD and maintenance hemodialysis, detailing the clinical focus, primary interventions, and regional characteristics that define renal nutrition research globally.

### 3.3. Methodological and Population Heterogeneity

**Figure 3:**
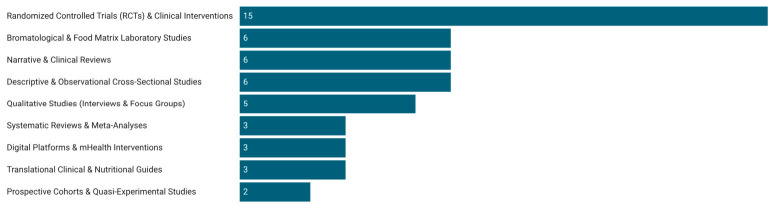
Methodological classification and distribution of the included literature (*n* = 49), categorizing the evidence by primary research design and clinical-behavioral focus.

Regarding the methodological architecture of the synthesized evidence, the screening and refinement procedure yielded a consolidated corpus of 49 definitive studies and exhibited a diverse range of research designs. Clinical experimentation represents a predominant pillar, with randomized controlled trials evaluating direct behavioral and dietary interventions in hospital and clinic settings. Observational, descriptive cross-sectional, and qualitative designs comprehensively capture the psychosocial barriers, food insecurity experiences, and health literacy limitations that condition patient self-management. Additionally, translational clinical guidelines and bromatological food-matrix analysis frameworks provide the cross-cutting theoretical support required to validate practical adaptation techniques. The systematic breakdown of these methodological typologies is structured and detailed in Figure 3.

The cross-analysis between geographical regions and study types reveals how distinct continents approach research on dietary self-management in advanced CKD (Table 4), an alignment heavily influenced by healthcare resources, clinical infrastructure, and regional socioeconomic characteristics [37].

Europe, Asia, and the Middle East fundamentally lead in clinical experimentation [38,39]. In Europe, clinical trials and analytical interventions typically focus on electronic health systems, web-based self-monitoring portals, or digital e-Health platforms [38,40], while concurrently driving the bromatological validation of thermal food processing techniques [38,40,41,42,43].

Conversely, the extensive clinical trials distributed across Asia and the Middle East focus predominantly on testing the efficacy of structured behavioral and interactive methods applied directly within dialysis centers [44,45]. These models center on optimizing intra-dialytic self-care through tools such as the teach-back pedagogical method [39], digital messaging networks (e.g., WeChat v4.1.11 [46]), or group psychoeducational programs [39,46,47].

North America’s focus, by contrast, shifts markedly toward observational and qualitative paradigms [13,48]. Rather than prioritizing large-scale clinical experimentation, the literature from the United States and Mexico largely consists of descriptive cross-sectional designs and qualitative/mixed-methods frameworks [13,48]. This region prioritizes establishing in-depth situational diagnoses, critically evaluating food insecurity, low health literacy, and socioeconomic inequalities [48,49], alongside assessing overall diet quality via established tools (such as the Healthy Eating Index) before implementing resource-intensive dietary interventions [48,49].

Developing economies and resource-constrained environments excel in practical, culturally tailored adaptations [37]. In regions where funding for large-scale randomized clinical trials is restricted, development reports, narrative clinical reviews, and targeted quasi-experimental frameworks adapted to local realities stand out [50]. Here, investigators prioritize immediate practical approaches, utilizing analog, text-free visual learning aids such as the South African ‘Renal Plate’ infographic [51] or validating behavior change theories (such as the Transtheoretical Model) within populations facing literacy barriers [51,52]. Finally, Oceania’s contribution highlights a distinct commitment to structured self-management support, leveraging decentralized interventions, digital media training, and tailored educational packages to translate complex renal guidelines into practical domestic skills [53,54,55,56,57].

**Table 4:** Research Approaches to CKD Dietary Self-Management by Geographic Region.

Geographic Region	Main Methodological Approach	Intervention Characteristics and Focus of Study	Authors
Europe	Clinical experimentation and bromatological validation	Use of electronic health systems, web-based self-monitoring portals, and e-Health platforms. Clinical validation of thermal food processing (cooking techniques).	[38,39,40,41,42,43,44,46,47]
Asia and the Middle East	Behavioral and interactive clinical trials	Interventions applied directly within dialysis centers. Use of the teach-back pedagogical method, digital messaging networks (e.g., WeChat, Telegram), and group psychoeducation.	[39,45,46,47]
North America (USA and Mexico)	Observational, cross-sectional designs, and qualitative/mixed-methods	Establishment of in-depth situational diagnoses. Evaluation of food insecurity, low health literacy, socioeconomic disparities, and overall diet quality (e.g., Healthy Eating Index).	[13,48,49]
Developing Economies (e.g., South Africa, Brazil)	Development reports, narrative reviews, and quasi-experimental designs	Practical and culturally tailored adaptations in resource-constrained settings. Creation of analog text-free visual learning aids (e.g., “Renal Plate”) and application of behavior change models (e.g., Transtheoretical Model).	[37,50,51,52]
Oceania (Australia)	Clinical trials for structured self-management support	Decentralized interventions focused on the home environment. Telehealth, telephone coaching, SMS, and tailored educational packages designed to translate complex guidelines into practical skills.	[53,54,55,56,57]

### 3.4. Thematic Mapping Using the WHO ICF Framework

The structuring of the literature using the International Classification of Functioning, Disability and Health (ICF) reveals that nutrition in CKD transcends simple food intake, involving a complex network of biological, behavioral, and social factors (Table 5).

#### 3.4.1. Body Functions (b)

The physiological alterations of CKD dictate the strict nutritional requirements of patients. General metabolic functions (b540) are profoundly affected, necessitating a delicate balance to prevent the accumulation of electrolytes, uremic toxins, and disorders of mineral metabolism [44,58,59]. As a consequence of disease progression and severe dietary restrictions, weight maintenance functions (b530) are impaired, increasing the risk of malnutrition and protein-energy wasting [60].

Additionally, patients face alterations in gustatory function (b250), such as dysgeusia and a loss of food palatability, which complicate nutritional intake [60,62]. Regarding mental health, energy functions (b130) decrease notably, manifesting as severe episodes of fatigue and lack of appetite or uremic anorexia [44,63]. Likewise, emotional functions (b152) are compromised, with constant feelings of fear, guilt, frustration, and depression emerging due to the burden of dietary restrictions and their effects on lifestyle [13,64,73].

#### 3.4.2. Activities and Participation (d)

Autonomous diet management places high demands on learning and applying knowledge. Patients need to develop their health literacy (d155) and learn to read (d145) to understand the fundamentals of the renal diet, properly interpret nutritional labels, and identify harmful hidden phosphorus additives in commercial products [44,46,65,66,68,69]. Within the domain of domestic life, preparing meals (d630) represents an unceasing practical challenge; patients and their caregivers must master specific culinary skills, such as wet cooking techniques and prolonged soaking, to effectively reduce mineral loads (such as potassium and phosphorus) without sacrificing essential nutrients [41,42,46,49,51].

At the self-care level, eating (d550) involves a rigorous modification of intake patterns, requiring portion control and constant vigilance that exhausts the patient [13,37,61,70]. Finally, diet drastically impacts community and social life (d910, d920). Normal commensality is fractured, creating significant barriers to dining at restaurants and causing a sense of isolation and separation from friends and family due to the inability to consume the same foods during social gatherings [64,74].

**Figure 4:**
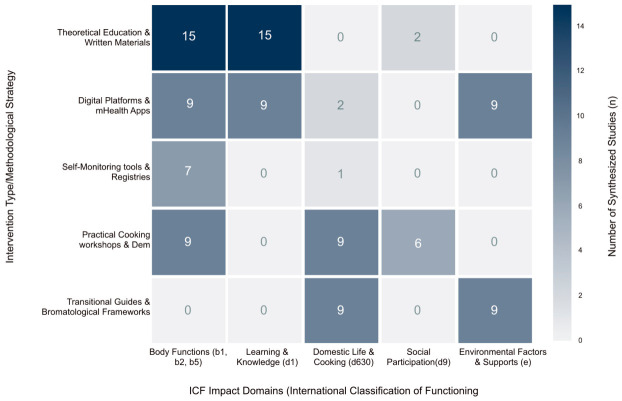
Evidence and gap map of the included literature (*n* = 49) cross-referencing intervention types against international classification of functioning, disability, and health (ICF) impact domains.

The matrix reveals a polarized research landscape. While clinical literature heavily saturates patient education through theoretical and cognitive domains, a profound evidence gap persists regarding practical, home-based culinary execution (*d*630). Active cooking workshops and translational bromatological frameworks emerge as the only methodology capable of bridging this gap, addressing not only metabolic control but also mitigating social isolation and preserving patient commensality (Figure 4).

#### 3.4.3. Environmental Factors (e)

The patient’s physical and social environment acts simultaneously as both a barrier and a facilitator. Support from immediate family members (e310) is a fundamental component for achieving dietary adherence; however, preparing separate meals or adapting the entire household’s diet often generates an enormous workload and significant stress for the primary caregiver [13,46]. Similarly, the role of health professionals (e355) is vital, as receiving individualized nutritional counseling and structured education from physicians and dietitians fosters empowerment and significantly improves clinical outcomes [28,47,75].

Conversely, the economic situation and assets (e165) represent a decisive barrier. Poverty and a lack of financial resources critically limit the patient’s ability to acquire the fresh foods required by the diet [13,37,51]. This directly interacts with products or substances for consumption (e110), forcing the patient to navigate a food environment saturated with harmful ultra-processed products and to attempt to adapt clinical guidelines to their own culture and local availability [74,76]. Finally, products and technology for communication (e125) have proven to be powerful modern facilitators, utilizing digital health interventions, telemedicine, SMS coaching reminders, and interactive intradialytic videos to simplify education and encourage healthy habits in real-time [39,40,54,72].

### 3.5. Evidence According to Study Context

The evidence shows that the hospital setting (intradialytic) is utilized to conduct structured interventions that do not require additional time from the patient (e.g., videos during the 4 h dialysis sessions or the teach-back method). In contrast, the out-of-hospital setting seeks to extend dietary self-management into daily life through telehealth, food diary logging in mobile applications, home-based family support, and virtual groups. This approach allows for real-time support when patients face the daily culinary and consumption dilemmas encountered at home (Table 6).

### 3.6. Facilitators and Barriers to Dietary Adherence in CKD

The analysis of barriers and facilitators through the framework of the International Classification of Functioning, Disability and Health (ICF) reveals that adherence to the renal diet is a phenomenon highly conditioned by the patient’s environment and resources.

Economic limitation is the most profound structural obstacle to dietary adherence in CKD. Poverty and the high cost of fresh foods force low-resource patients to rely on charity packages or ultra-processed foods, which makes strict follow-up of medical prescriptions impossible [13,37]. At the domestic level, the complexity of the required culinary techniques—such as prolonged soaking or double boiling to reduce minerals—generates an excessive workload, lack of time, and exhaustion, especially when the caregiver must prepare separate meals for the patient and the rest of the family [48,74].

Furthermore, the renal diet drastically alters the patient’s commensality and social life. Individuals experience moral dilemmas and a strong sense of isolation when refusing food offered by family or friends, feeling that their new social identity and restrictions separate them from their community [64]. This social disconnection adds to an enormous emotional burden characterized by frustration, aversion toward food perceived as monotonous or tasteless, and constant guilt regarding cravings [13,64]. Additionally, receiving fragmented or irrelevant medical advice that does not adapt to the patient’s culture aggravates this frustration, frequently leading to a lack of discipline.

However, to counteract these barriers, social support and adapted educational methodologies are fundamental pillars. Support from immediate family members in purchasing and preparing food significantly facilitates adherence, preventing diet abandonment by reducing the direct burden on the patient [13]. In the clinical setting, nutritional education proves to be a powerful facilitator when it is individualized and practical. The use of text-free visual analogies, such as the “Renal Plate” used in low-literacy settings, and the application of the teach-back method guarantee knowledge assimilation and foster self-efficacy [39,51]. Likewise, patients’ internal motivation flourishes when they perceive direct clinical improvements or symptom relief, acting as a driver to maintain adherence [13].

Finally, digital health (e-Health) interventions and telehealth have emerged as innovative tools to overcome barriers in the out-of-hospital environment. The use of food diaries in web applications, intensive telephone coaching, and automated text message (SMS) reminders allow for real-time guidance of patient decision-making, sustaining motivation and facilitating the resolution of culinary dilemmas at home [38,40,55] (Table 7).

## 4. Discussion

This scoping review was developed with the objective of mapping and synthesizing current evidence in response to the question formulated through the PCC framework: to evaluate how, in the Population of patients with CKD, the Concept of nutritional requirements and metabolic control intersects with the acquisition of health literacy and culinary skills, framed within a global context that integrates clinical, community, and home environments.

Before synthesising the available evidence, a fundamental caveat must be stated explicitly: the current literature does not yet provide a robust, direct empirical link between the three mapped constructs in the CKD population. Only a limited subset of the 49 included studies was specifically designed to investigate the intersection of dietary knowledge, food literacy, and practical culinary skills simultaneously, and none establish a causal pathway from food literacy to measurable clinical outcomes via culinary skill acquisition. This evidence gap is itself a primary finding of this scoping exercise. The synthesis that follows should therefore be read as a map of what has been investigated and where the critical voids lie—not as confirmation of a well-evidenced clinical model.

Against this backdrop, it is necessary to clarify the operational definitions of the three core constructs examined in this review, given the terminological heterogeneity identified across the included studies. Dietary knowledge (DK) or nutrition knowledge (NK) refers to the declarative, theoretical understanding of nutrient content, dietary restrictions, and clinical guidelines [79], the cognitive dimension of knowing what to eat or avoid. Dietary knowledge (DK) is considered as one of the factors affecting food intake [80] and has been reported to be positively associated with diet quality [81,82].

Food literacy, a broader and more encompassing construct, integrates the functional, interactive, and critical competencies required to plan, select, prepare, and evaluate food within a specific health context [83]; it encompasses both cognitive and behavioral dimensions and has been operationalized in CKD research through validated instruments such as food frequency questionnaires, dietary recall interviews, and health literacy scales. Culinary skills, the most practically oriented of the three, refer to the technical and procedural competencies applied during food preparation, including the ability to select appropriate cooking methods, adapt recipes to renal dietary requirements, and apply mineral-reduction techniques (e.g., leaching, double boiling) [84,85]. While these three constructs are conceptually distinct, the evidence synthesized in this review consistently demonstrates that they function as an interdependent continuum: dietary knowledge is a necessary but insufficient precondition for food literacy, which in turn provides the cognitive scaffolding upon which practical culinary competencies are built and sustained.

The included studies suggest that dietary management in CKD cannot be understood solely as a prescription of restrictions, but rather as a process of supporting self-care that integrates nutritional education, individualization, behavioral support, and adaptation to the patient’s life context [13]. This perspective aligns with proposed biopsychosocial and multidisciplinary approaches to chronic kidney disease, especially in advanced stages [5,53,86]. It is essential to acknowledge that the dietary self-management landscape differs fundamentally between patients on maintenance hemodialysis (HD) and those managed conservatively at CKD stages 3–5, and that conflating these two populations risks obscuring clinically meaningful distinctions [87]. Patients undergoing HD face a life profoundly disrupted by treatment schedules—typically three sessions per week of three to four hours each—which impose rigid temporal, social, and logistical constraints on meal planning, food acquisition, and culinary practice. Their dietary restrictions are particularly stringent with respect to fluid, potassium, and phosphorus, as the intermittent nature of dialysis creates accumulation dynamics that demand strict inter-dialytic compliance [76,87,88,89,90,91]. Conversely, patients in CKD stages 3–5 under conservative management must navigate a sustained, low-protein or very-low-protein dietary prescription over a prolonged and often indefinite timeline, where the primary challenge is long-term behavioral consistency rather than acute inter-session restriction. Adhering to a Low Protein Diet (LPD) or Very Low Protein Diet (VLPD) presents unique and formidable challenges for these patients. The primary difficulty lies in the profound loss of meal palatability and the cultural disruption of eating habits, as animal proteins traditionally anchor the sensory profile (umami) and social commensality of conventional diets. Consequently, patients frequently struggle to maintain adequate total caloric intake, increasing the risk of protein-energy wasting (PEW). Furthermore, successfully substituting animal proteins with plant-based alternatives requires advanced culinary skills to build flavor profiles using herbs and spices, as patients must avoid the compensatory—and dangerous—use of high-sodium condiments or ultra-processed foods. The quality-of-life implications also diverge: conservative management patients retain greater autonomy over daily routines, which shapes both their barriers to and opportunities for culinary skill acquisition differently. The educational interventions and culinary strategies reviewed here must therefore be interpreted within the specific clinical context to which they apply, and future research should systematically distinguish between these populations when designing, implementing, and evaluating nutritional interventions.

From a pathophysiological perspective, the progression of CKD across the CKD3b, CKD5, and CKD5D spectrum severely compromises systemic homeostasis, resulting in the progressive inability to regulate mineral balance, acid–base equilibrium, and uremic toxin excretion [44,58]. To manage this metabolic load, official clinical guidelines—such as the KDOQI practice updates—impose rigorous numerical restrictions on protein, sodium, potassium, and phosphorus. Within this strictly regulated framework, food literacy and culinary skills do not substitute clinical guidelines; instead, they function as the indispensable operational bridge to implement them safely at home—a concept that a subset of the included literature has characterised, rhetorically, as an ‘external artificial kidney’ [69]. The systematic application of certified cooking techniques, such as prolonged soaking and double-boiling with water disposal, constitutes an evidence-based mechanism to fulfill regulated targets, achieving a drastic reduction in mineral bioavailability before ingestion without inducing protein-energy wasting [42,43,59,69,92]. This implies that a patient with high health literacy manages to mitigate the solute load entering the body, assuming proactive control of their internal metabolic balance [46]. The clinical relevance of these culinary strategies rests on concrete bromatological evidence: The clinical management of chronic kidney disease (CKD) requires precise nutritional targets that dynamically adapt to the disease trajectory. In pre-dialysis stages, protein restriction is strictly maintained between 0.6 and 0.8 g/kg/day—or even reduced to 0.3–0.4 g/kg/day in very-low-protein diets (VLPDs) supplemented with ketoanalogues—to minimize renal workload; however, this requirement increases to 1.0–1.3 g/kg/day during dialysis to compensate for treatment-induced catabolism and prevent protein-energy wasting [37,58,60,61]. Concurrently, phosphorus intake must be rigidly restricted to 800–1000 mg/day, with advanced stages often requiring limits below 400–600 mg/day to maintain mineral homeostasis and prevent cardiovascular complications [44,58,59,69]. To achieve these delicate balances without compromising protein adequacy, the literature strongly supports the implementation of plant-dominant diets (PLADO) and specific culinary skills. Since plant-based phosphorus (phytate) has a significantly lower bioavailability (20–50%) compared to the highly bioavailable inorganic additives found in ultra-processed foods (~100%), sourcing at least 50% of dietary protein from plants offers a crucial metabolic advantage [50,58,68,69]. Furthermore, the application of wet-cooking techniques, such as prolonged soaking and boiling, effectively reduces the phosphorus load of foods by 27% to 50% without altering their essential protein content, acting as a practical strategy for patients to control their dietary intake at home [41,42,43,69].

In this context, available evidence shows that food literacy, self-efficacy, and practical food preparation skills are associated with better adherence and improved diet quality, supporting the need for broader educational interventions beyond simple dietary prescription [10,93,94]. While establishing a single pooled percentage of dietary adherence post-intervention is methodologically unfeasible due to the profound heterogeneity of measurement tools across the included studies—which range from biochemical markers (such as serum phosphorus and 24 h urinary sodium) to self-reported psychometric scales and the Healthy Eating Index—the synthesized evidence demonstrates a consistent, significant improvement. Studies that explicitly integrated practical culinary training and food literacy reported substantial enhancements in these adherence proxies. This contrasts starkly with the 20% to 70% non-adherence rates typically observed under conventional, theory-only dietary counseling, confirming that practical skill acquisition is a primary driver of sustained dietary compliance. Regarding these claims, the current review shows that despite the clinical efficacy of nutritional therapy, its adherence is heavily contingent upon the social determinants of health and the individual’s psychosocial environment. Qualitative and mixed-methods literature highlights that the renal diet profoundly alters commensality, generating feelings of social isolation, guilt, moral dilemmas, and frustration in patients who are unable to participate fully in family eating dynamics [64].

Available evidence suggests that educational interventions in CKD are transitioning from asymmetrical, hospital-centric models toward strategies more focused on the patient and their home environment. In the in-hospital and intra-dialysis settings, the implementation of structured theoretical models—such as the “teach-back” pedagogical method, the use of simulations via decision trees, and multimodal or video-based psychoeducation—has demonstrated superior efficacy in improving self-efficacy, knowledge assimilation, and the reduction in symptom burden [39,44,67,72]. Along the same lines, various studies show an increase in comprehension, self-efficacy, and quality of life, alongside a decrease in symptom burden [95,96]. Beyond the intra-dialytic setting, it is important to acknowledge the broader landscape of in-person intervention opportunities that remain underexplored in the current literature. Multidisciplinary nutrition teams—comprising registered dietitians, nephrology nurses, and social workers—embedded within nephrology outpatient clinics represent a high-impact point of contact for patients in CKD stages 3–5 who are not yet on dialysis and may be more receptive to behavioral change. Primary care physician appointments equally constitute an underutilized venue for early nutritional screening and culinary skills referral, particularly given that most CKD patients in earlier stages are managed in primary care settings rather than specialist nephrology units. Incorporating structured dietary literacy assessments and brief culinary medicine counselling into routine primary care consultations for CKD could extend the reach of these interventions substantially, particularly in healthcare systems where access to specialist nephrology dietitians is limited. Furthermore, community pharmacists, who have frequent contact with CKD patients for medication management, represent an additional underutilized touchpoint for reinforcing dietary and culinary guidance. This diversification of delivery channels is essential to ensure equitable access to nutritional education across healthcare systems with varying levels of specialist infrastructure.

In response to the objectives of this scoping review, the localized evidence underscores that the optimization of clinical parameters in CKD inexorably requires improving patients’ health literacy and culinary skills. However, for these interventions to be effective and sustainable, healthcare professionals must abandon the prescriptive approach based on generic restrictions and move toward a model of individualized nutritional education that is empathetic and adapted to the patient’s psychosocial, economic, and technological context.

Future Research Priorities. To address the empirical gaps identified in this review, future research should prioritise large-scale, cross-sectional survey studies specifically designed and validated to measure the concurrent levels of food literacy and practical culinary skills in the CKD population. These surveys should employ standardised instruments capable of investigating whether higher cognitive food literacy directly correlates with superior procedural cooking competencies at home across diverse demographic cohorts. Establishing this statistical baseline relationship is a prerequisite for designing targeted, scalable culinary medicine interventions. Longitudinal and interventional designs will subsequently be needed to test whether improvements in culinary competence translate into measurable gains in dietary adherence and clinical biomarkers. Future studies should also systematically distinguish between HD and CKD 3–5 conservative management populations, given the fundamentally different dietary demands of each group.

### Limitations of the Review

The interpretation of the results of this scoping review should be made considering its inherent methodological limitations. First, the methodological heterogeneity of the included studies—in terms of design, population, measurement instruments, and operationalization of key concepts—limits direct comparability between findings and hinders the extraction of uniform conclusions regarding the efficacy of specific interventions. This heterogeneity is, however, an expected and acceptable characteristic in the context of a scoping review, whose objective is precisely to map the breadth and diversity of available evidence rather than to synthesize comparable results.

Second, variability in the operational definition of terms such as “dietary literacy,” “culinary skills,” and “adherence” among the included studies complicates conceptual synthesis and may have influenced both the inclusion and classification of some records. The absence of a consensus taxonomy for these concepts in the field of CKD is itself a relevant finding of the review, reinforcing the need for conceptual clarification work prior to the development of new interventions.

Third, the search was limited to four electronic databases and publications in English and Spanish, which may have excluded relevant studies published in other languages or indexed in sources not consulted, such as regional databases (LILACS, SciELO, CNKI) or relevant gray literature. This limitation is particularly significant concerning the identified geographical gap, as studies conducted in non-Anglophone contexts may be underrepresented in the international databases consulted.

## 5. Conclusions

This scoping review maps the existing evidence at the intersection of nutritional requirements, dietary knowledge, and culinary skills in the adult CKD population. The primary finding is that this intersection remains substantially understudied: the included literature is heterogeneous in design, population, and measurement, and no included study was designed to establish a direct empirical link between food literacy and practical culinary competence in CKD. The three constructs are conceptually distinct and likely interdependent, but their precise interaction and its impact on clinical outcomes have not yet been demonstrated with sufficient methodological rigour.

What the available evidence does confirm is that dietary non-adherence in CKD is multidimensional—shaped by social determinants, economic constraints, emotional burden, and the gap between theoretical knowledge and practical meal preparation—and that interventions addressing only the cognitive dimension of dietary knowledge are insufficient. Educational strategies that incorporate practical culinary components and are adapted to the patient’s social and cultural context appear associated with improved adherence proxies, though the heterogeneity of measurement instruments precludes firm conclusions about efficacy.

The principal contribution of this review is therefore the mapping of a precise, empirically grounded research agenda. Future primary research should prioritise validated, large-scale studies capable of characterising the relationship between food literacy and culinary skill acquisition across diverse CKD demographic groups, thereby providing the foundation necessary to design and evaluate scalable culinary medicine interventions.

## Figures and Tables

**Table 1:** Research question breakdown following the PCC framework.

Population (P)	Concept (C)	Context (C)
Patients with Chronic Kidney Disease (CKD) in advanced stages (3, 4, and 5) and those on renal replacement therapy (dialysis).	Multidimensional interaction between specific nutritional requirements, the level of dietary knowledge, and culinary skills (food literacy).	Healthcare settings, encompassing both the clinical environment (hospitals, nephrology units) and the community environment (home, primary health centers).

**Table 2:** Search strategy.

Data Sources	Initial	Title/Abstract	Full-Text
Pubmed	246	35	27
EBSCO	47	27	18
WoS	6	3	2
Scopus	318	0	0
Snowballing			2

NOTE: The final inclusion of 0 records from Scopus does not imply an absence of relevant literature within this database. Due to the extensive overlap between indexing systems, all eligible studies identified in Scopus were primary duplicates of records already captured and retrieved through PubMed/MEDLINE and EBSCO during the deduplication phase. To maintain procedural traceability, these duplicates were systematically allocated to their primary biomedical database source.

**Table 3:** Types of Study Interest by Geographical Region.

Geographical Region	Included Countries	Aggregated Sample Size (*N*) *	Primary Study Focus and Clinical Applications
East and Southeast Asia	China, Japan, Taiwan, South Korea, Malaysia, Singapore	1155	Implementation of structured behavioral methods (e.g., teach-back, mHealth via WeChat) directly within hemodialysis clinics. Analysis of traditional dietary adaptations (e.g., low-protein rice).
Middle East	Iran, Saudi Arabia, Turkey, Jordan	812	Population-based assessment of dietary literacy regarding potassium and phosphorus. Video-assisted training and interactive digital education for intra-dialytic symptom control.
North America	USA, Mexico	186	Socioeconomic inequalities, food insecurity, and low health literacy diagnostics. Evaluation of overall diet quality (HEI) and safety of plant-dominant low-protein patterns (PLADO).
Europe	Netherlands, UK, Spain, Italy, Denmark, Croatia, Slovenia, Cyprus	159	Web-based e-Health platforms for sodium self-monitoring. Bromatological and clinical validation of thermal processing techniques (boiling/leaching) to reduce mineral bioavailability.
South America and Africa	Brazil, South Africa	63	Behavior change models (Transtheoretical Model) for serum phosphorus control in low-resource environments. Development of culturally adapted, text-free visual learning tools (e.g., “Renal Plate”).
Oceania	Australia	35	Phenomenological exploration of patient perception regarding contradictory fluid and mineral guidelines, and self-management support.
Global Consensus	24 Countries	2410 patients	Consolidated Clinical Sample (Adults with advanced CKD stages 3–5 under conservative care or maintenance hemodialysis).

* Note: Methodological consensus frameworks, bromatological food matrix analysis studies, and narrative clinical guidelines included in Section C of the Supplementary Materials are synthesized within the discussion of this review but are excluded from the direct patient sample aggregation to prevent epidemiological duplication.

**Table 5:** Distribution of Studies According to WHO ICF Codes.

ICF Category	ICF Subcategory	Application/Phenomenon in CKD	Authors
Digestive, metabolic, and endocrine system functions (b5)	General metabolic functions (b540)	Alteration of homeostasis (mineral and acid–base imbalance) and inability to manage metabolic load (accumulation of uremic toxins and nitrogen).	[44,58,59]
	Weight maintenance functions (b530)	Malnutrition and protein-energy wasting secondary to disease and diet.	[60,61]
Sensory functions and pain (b2)	Gustatory function (b250)	Taste alterations, loss of food palatability, and dysgeusia.	[60,62]
Mental functions (b1)	Energy and drive functions (b130)	Reduced energy levels, fatigue, and lack of appetite (uremic anorexia).	[44,63]
	Emotional functions (b152)	Fear, guilt, frustration, depression, and negative attitudes toward restrictions.	[13,46,64]
Learning and applying knowledge (d1)	Acquisition of skills (d155)	Health literacy, comprehension, and assimilation of renal diet knowledge.	[46,65,66,67]
	Learning to read (d145)	Reading, interpretation, and understanding of nutritional labels and hidden additives.	[68,69]
Domestic life (d6)	Preparing meals (d630)	Culinary skills, planning, and wet cooking/soaking techniques.	[41,42,43,51,69]
Self-care (d5)	Eating (d550)	Adherence to the renal diet, portion control, and modification of intake patterns.	[13,37,61,70]
Community, social, and civic life (d9)	Community life/Recreation and leisure (d910/d920)	Commensality, social impact, dining out, and feelings of isolation.	[48,64,71]
Support and relationships (e3)	Immediate family members (e310)	Fundamental family support for adherence or caregiver burden for the person cooking.	[13,48]
	Health professionals (e355)	Interventions, counseling, and structured education by dietitians and physicians.	
Products and technology (e1)	Assets (e165)	Patient’s economic situation, poverty, and financial limitations for food purchasing.	[13,37,51]
	Products or substances for personal consumption (e110)	Availability of fresh vs. ultra-processed foods, and cultural adequacy.	[50,68]
	Products and technology for communication (e125)	Use of apps, telemedicine, web diaries, SMS coaching, and educational videos.	[39,40,54,55,72]

**Table 6:** Intervention Distribution According to Setting.

Study Setting	Type of Interventions and Approaches Implemented	Authors
Hospital (Hemodialysis/Peritoneal Dialysis Centers)	Education during treatment: Utilization of machine connection time to apply video-assisted education (with individual headphones) for symptom control. On-site culinary skills: Provision of hospital meals prepared using boiling techniques to demonstrate the palatability of demineralized foods. Behavioral models: Application of the Teach-back method to ensure information retention. Use of clinical decision trees and analog visual tools (e.g., “Create a Renal Plate” game) adapted to literacy levels. Fatigue management: Education for energy conservation during dialysis.	[39,42,43,44,51,52,56,57,63,67,72]
Hospital (Nephrology Outpatient Clinic and Primary Care)	Multidisciplinary Programs: Face-to-face empowerment modules, goal setting, and counseling sessions with dietitians and physicians. Dietary pattern shifts: Guided counseling on Mediterranean diet principles for peritoneal dialysis patients and prescription of conventional self-care interventions. Diagnostic Assessment (Observational): Face-to-face interviews and focus groups in the clinic to evaluate socioeconomic barriers and health literacy, and to conduct food frequency questionnaires.	[13,45,49,52,53,62,63,73,77]
Out-of-Hospital (Home-based and Telehealth)	Digital Health Interventions (e-Health): Use of interactive web platforms and applications (e.g., *My Kidneys & Me* and the *SUBLIME* app) so that patients can maintain an online food diary and record their blood pressure at home autonomously. Remote coaching: Intensive telehealth interventions through scheduled telephone calls and the regular sending of personalized text messages (SMS) to motivate renal diet adherence.	[38,40,53,54,55]
Out-of-Hospital (Virtual Environment, Social Media, and Community)	Comprehensive Virtual Education: Implementation of the “Jumpstart” nutrition program focused on plant-based diets, delivered entirely via the Zoom platform. Social media and E-learning: Distribution of short videos on dietary techniques and continuous group support using commercial messaging apps (such as WeChat and Telegram) to reach patients in their daily lives. Family environment: Face-to-face group education conducted directly in the patient’s home to involve their social and family network. Population Assessment: National surveys (e.g., KNHANES) to evaluate overall healthy eating indices.	[45,46,59,70,78]

**Table 7:** Barriers and Facilitators for Dietary Adherence in Chronic Kidney Disease Structured According to ICF Domains.

Type	Category (ICF Domain)	Specific Factor	Description and Impact
Barrier	Products and technology: Assets (e165)	Economic limitations	Poverty and the high cost of fresh foods act as the primary obstacle to following the renal diet. In vulnerable populations, high unemployment rates and dependency on food pantries or charity food packages make compliance impossible. This economic limitation drastically dictates the patients’ ability to choose.
	Domestic life: Preparing meals (d630)	Complexity, effort, and lack of time	The preparation of renal-friendly foods requires complex techniques and the cooking of multiple separate meals for the patient and the rest of their family. This generates frustration, caregiver exhaustion, and a lack of time due to work schedules or dialysis sessions. Furthermore, patients report great difficulty in estimating portions and additives in restaurants.
	Community, social, and civic life: Recreation and leisure (d920)	Social isolation and altered commensality	Diets severely restrict participation in gatherings and family meals. Refusing food offered by loved ones causes conflict, guilt, and a strong moral dilemma, often leading the patient to break the diet to fit in or avoid appearing ungrateful.
	Mental functions: Emotional functions (b152) and Gustatory function (b250)	Food aversion and emotional burden	Patients experience feelings of deprivation, anger due to food monotony, and rejection of the renal diet, considering it “bland” or tasteless. The fear that certain foods will worsen their condition and the guilt of having “cravings” or desiring high-potassium foods also hinder adherence.
	Support and relationships: Health professionals (e355)	Fragmented, generic, or contradictory advice	Receiving confusing information, an overload of instructions, or irrelevant guidelines not adapted to the patient’s culture generates frustration. Often, counseling focuses strictly on restrictions, leading to a loss of patient control and discipline.
Facilitator	Support and relationships: Health professionals (e355) and Learning (d155)	Individualized and practical nutritional education	When the dietary prescription is flexible, includes replacement alternatives, and is adapted to the patient’s preferences and literacy, knowledge assimilation improves. The use of visual analogies (such as the “renal plate” or color-coded traffic lights) and the teach-back method strongly foster self-efficacy.
	Support and relationships: Immediate family members (e310)	Social and family support	Having committed family members who support food shopping, planning, and preparation is an essential pillar that reduces diet abandonment by decreasing the direct burden on the patient.
	Mental functions: Emotional functions (b152)	Internal motivation and pursuit of clinical benefits	The patient’s readiness for change is enhanced when they perceive direct health benefits. The motivation to avoid medical catastrophes, control electrolyte levels, and alleviate uremic symptoms or fatigue is a strong driver for maintaining long-term discipline.
	Products and technology for communication (e125)	e-Health and telehealth interventions	The use of digital tools such as mobile applications, intake trackers, virtual diaries, short informative videos, and personalized text messages (SMS) helps keep the patient motivated. These interventions guide culinary decision-making in “real-time” and overcome the barrier of traveling to the clinic.

## Data Availability

No new data were created or analyzed in this study. Data sharing is not applicable to this article.

## Supplementary Materials

The following supporting information can be downloaded at: https://www.mdpi.com/article/10.3390/nu18142245/s1, The studies finally included, along with all their characteristics, can be found in the Supplementary Materials.

## References

[1] Bikbov B., Purcell C., Levey A.S., Smith M., Abdoli A., Abebe M., Adebayo O.M., Afarideh M., Agarwal S.K., Agudelo-Botero M. (2020). Global, Regional, and National Burden of Chronic Kidney Disease, 1990–2017: A Systematic Analysis for the Global Burden of Disease Study 2017. Lancet.

[2] Kramer H. (2019). Diet and Chronic Kidney Disease. Adv. Nutr..

[3] Rhee C.M., Wang A.Y.M., Biruete A., Kistler B., Kovesdy C.P., Zarantonello D., Ko G.J., Piccoli G.B., Garibotto G., Brunori G. (2023). Nutritional and Dietary Management of Chronic Kidney Disease Under Conservative and Preservative Kidney Care Without Dialysis. J. Ren. Nutr..

[4] Ikizler T.A., Burrowes J.D., Byham-Gray L.D., Campbell K.L., Carrero J.J., Chan W., Fouque D., Friedman A.N., Ghaddar S., Goldstein-Fuchs D.J. (2020). KDOQI Clinical Practice Guideline for Nutrition in CKD: 2020 Update. Am. J. Kidney Dis..

[5] Palmer S.C., Maggo J.K., Campbell K.L., Craig J.C., Johnson D.W., Sutanto B., Ruospo M., Tong A., Strippoli G.F.M. (2017). Dietary Interventions for Adults with Chronic Kidney Disease. Cochrane Database Syst. Rev..

[6] Kalantar-Zadeh K., Mattix-Kramer H.J., Moore L.W. (2021). Culinary Medicine as a Core Component of the Medical Nutrition Therapy for Kidney Health and Disease. J. Ren. Nutr..

[7] Shanmugapriya K., Yuvaraj S., Vishnupriya D., Vinitha K., Vijayanila G., Zamrun Begam T., Veeralakshmi M., Thilagavathi V., Vejaiyan R., Thanasekar R. (2024). Assessment of Knowledge on Dietary Management of Chronic Kidney Disease Among Patients Undergoing Hemodialysis at a Tertiary Care Hospital in South India: A Cross-Sectional Analytical Study. Cureus.

[8] Norouzi S., Liu K.S., Bustamante E., La T., Mitch W.E., Pivert K., Staggers K.A., Shusterman B., Yuan C.M., Raghavan R. (2021). The Kidney Diet Challenge: An Experiential Educational Experience. Kidney360.

[9] (2022). Nutrition Guideline for Professional Reference Only Renal. https://www.albertahealthservices.ca/assets/info/nutrition/if-nfs-ng-renal.pdf.

[10] Duan D.F., Liu M., Chen Y., Huang Y.Y., Shi Y.Y. (2022). Food Literacy and Its Associated Factors in Non-Dialysis Patients with Chronic Kidney Disease in China: A Cross-Sectional Study. Patient Prefer. Adherence.

[11] Lambert K., Mullan J., Mansfield K. (2017). An Integrative Review of the Methodology and Findings Regarding Dietary Adherence in End Stage Kidney Disease. BMC Nephrol..

[12] Beto J.A., Schury K.A., Bansal V.K. (2016). Strategies to Promote Adherence to Nutritional Advice in Patients with Chronic Kidney Disease: A Narrative Review and Commentary. Int. J. Nephrol. Renov. Dis..

[13] Trigueros-Flores X.B., Luna-Hernández G., Santos-Lopez M.F., Pérez-Galván L., Flores-Camacho K.J., Díaz-Canchola L.M., Cueto-Manzano A.M., Chávez-Chávez H.E., Cerrillos-Gutiérrez J.I., Rojas-Campos E. (2025). Barriers and Facilitators to Adherence to a Healthy Diet Across the Spectrum of Chronic Kidney Disease. Patient Prefer. Adherence.

[14] Zhu S., Mohd Yusoff D., Yusoff H., Cheng K.Y., Feng X., Chen H. (2024). Knowledge, Attitude, and Practice Regarding Malnutrition amongst Patients with Chronic Kidney Disease in China: A Qualitative Study. J. Educ. Health Promot..

[15] Bross R., Noori N., Kovesdy C.P., Murali S.B., Benner D., Block G., Kopple J.D., Kalantar-Zadeh K. (2010). Dietary Assessment of Individuals with Chronic Kidney Disease. Semin. Dial..

[16] Nogueira-Rio N., Mondragon Portocarrero A.d.C., Lamas Freire A., Franco C.M., Canbolat A.A., Karav S., Miranda Lopez J.M. (2025). Rethinking Nutrition in Chronic Kidney Disease: Plant Foods, Bioactive Compounds, and the Shift Beyond Traditional Limitations: A Narrative Review. Foods.

[17] Lim J.H., Chinna K., Khosla P., Daud Z.A.M., Karupaiah T. (2020). Understanding How Nutrition Literacy Links to Dietary Adherence in Patients Undergoing Maintenance Hemodialysis: A Theoretical Exploration Using Partial Least Squares Structural Equation Modeling. Int. J. Environ. Res. Public. Health.

[18] Chan C.H., Conley M., Reeves M.M., Campbell K.L., Kelly J.T. (2021). Evaluating the Impact of Goal Setting on Improving Diet Quality in Chronic Kidney Disease. Front. Nutr..

[19] Lockwood G., Davey L., McFarlane C., Gray N.A., Wright H.H. (2024). Factors Influencing Meal Provision and Dietary Support Behaviour of Caregivers of People with Chronic Kidney Disease: A Cross-Sectional Study. Nutrients.

[20] Torreggiani M., Avesani C.M., Contzen B., Cupisti A., Czaja-Stolc S., D’Alessandro C., Garneata L., Gutiérrez A., Lippi F., Mocanu C.A. (2025). Dos and Don’ts in Kidney Nutrition: Practical Considerations of a Panel of Experts on Protein Restriction and Plant-Based Diets for Patients Living with Chronic Kidney Disease. Nutrients.

[21] Sunwold D. (2007). Notes from the CKD Kitchen: Restaurant Dining. J. Ren. Nutr..

[22] Nutrition, Novel Foods and Food Allergens|EFSA. https://www.efsa.europa.eu/en/science/scientific-committee-and-panels/nda.

[23] Villalona S., Ortiz V., Castillo W.J., Garcia Laumbach S. (2021). Cultural Relevancy of Culinary and Nutritional Medicine Interventions: A Scoping Review. Am. J. Lifestyle Med..

[24] Kim S.M., Jung J.Y. (2020). Nutritional Management in Patients with Chronic Kidney Disease. Korean J. Intern. Med..

[25] Nooriani N., Mohammadi V., Feizi A., Shahnazi H., Askari G., Ramezanzade E. (2019). The Effect of Nutritional Education Based on Health Belief Model on Nutritional Knowledge, Health Belief Model Constructs, and Dietary Intake in Hemodialysis Patients. Iran. J. Nurs. Midwifery Res..

[26] (2015). Health Literacy Toolkit. For Low- and Middle-Income Countries. https://iris.who.int/server/api/core/bitstreams/41f18633-f9e2-4d7a-8ef3-399716ff8880/content.

[27] Hirsch I.B., Evert A., Fleming A., Gaudiani L.M., Guggenmos K.J., Kaufer D.I., McGill J.B., Verderese C.A., Martinez J. (2019). Culinary Medicine: Advancing a Framework for Healthier Eating to Improve Chronic Disease Management and Prevention. Clin. Ther..

[28] Schlueter R., Calhoun B., Harned E., Gore S. (2021). A VA Health Care Innovation: Healthier Kidneys Through Your Kitchen—Earlier Nutrition Intervention for Chronic Kidney Disease. J. Ren. Nutr..

[29] Martínez-Pineda M., Yagüe-Ruiz C., Vercet-Tormo A. (2020). Is It Possible to Include Potato in the Diet of Chronic Kidney Disease Patients? New Culinary Alternatives for Limiting Potassium Content. J. Ren. Nutr..

[30] Notaras S., Lambert K., Perz J., Makris A. (2022). Diet in the Management of Non-Dialysis Dependent Chronic Kidney Disease: Perceptions and Practices of Health Professionals. BMC Nephrol..

[31] Ebrahim Z., Glorieux G., Moosa M.R., Blaauw R. (2022). Effect of Simplified Dietary Advice on Nutritional Status and Uremic Toxins in Chronic Kidney Disease Participants. S. Afr. J. Clin. Nutr..

[32] D’Alessandro C., Piccoli G.B., Calella P., Brunori G., Pasticci F., Egidi M.F., Capizzi I., Bellizzi V., Cupisti A. (2016). “Dietaly”: Practical Issues for the Nutritional Management of CKD Patients in Italy. BMC Nephrol..

[33] Arksey H., O’Malley L. (2005). Scoping Studies: Towards a Methodological Framework. Int. J. Soc. Res. Methodol..

[34] Levac D., Colquhoun H., O’Brien K.K. (2010). Scoping Studies: Advancing the Methodology. Implement. Sci..

[35] Tricco A.C., Lillie E., Zarin W., O’Brien K.K., Colquhoun H., Levac D., Moher D., Peters M.D.J., Horsley T., Weeks L. (2018). PRISMA Extension for Scoping Reviews (PRISMA-ScR): Checklist and Explanation. Ann. Intern. Med..

[36] Papadakos J.K., Hasan S.M., Barnsley J., Berta W., Fazelzad R., Papadakos C.J., Giuliani M.E., Howell D. (2018). Health Literacy and Cancer Self-management Behaviors: A Scoping Review. Cancer.

[37] Ameh O.I., Cilliers L., Okpechi I.G. (2016). A Practical Approach to the Nutritional Management of Chronic Kidney Disease Patients in Cape Town, South Africa. BMC Nephrol..

[38] Hoekstra T., Dam M., Klaassen G., Bos W.J.W., Van Der Boog P.J.M., Vogt L., Van Jaarsveld B., Van Dijk S., Navis G., Meuleman Y. (2025). Self-Monitoring and Self-Efficacy in Patients with Chronic Kidney Disease During Low-Sodium Diet Self-Management Interventions: Secondary Analysis of the ESMO and SUBLIME Trials. Int. J. Behav. Med..

[39] Liu Y., Luo X., Ru X., Wen C., Ding N., Zhang J. (2024). Impact of a Multimodal Health Education Combined with Teach-Back Method on Self-Management in Hemodialysis Patients: A Randomized Controlled Trial. Medicine.

[40] Lightfoot C.J., Wilkinson T.J., Billany R.E., Sohansoha G.K., Vadaszy N., Ford E.C., Davies M.J., Yates T., Smith A.C., Graham-Brown M.P.M. (2025). Use, Usability, and Experience Testing of a Digital Health Intervention to Support Chronic Kidney Disease Self-Management: Mixed Methods Study. J. Med. Internet Res..

[41] Vrdoljak I., Panjkota Krbavčić I., Bituh M., Vrdoljak T., Dujmić Z. (2015). Analysis of Different Thermal Processing Methods of Foodstuffs to Optimize Protein, Calcium, and Phosphorus Content for Dialysis Patients. J. Ren. Nutr..

[42] Vrdoljak I., Panjkota Krbavčić I., Bituh M., Leko N., Pavlović D., Vrdoljak Margeta T. (2017). The Impact of Education and Cooking Methods on Serum Phosphate Levels in Patients on Hemodialysis: 1-year Study. Hemodial. Int..

[43] Vrdoljak I., Pozaić A., Bituh M., Leko N., Vrdoljak Margeta T., Pavlović D., Panjkota Krbavčić I. (2024). Education and Cooking Methods in the Management of Calcium and PTH Serum Levels in Patients on Hemodialysis: A Randomized Controlled Study. J. Nephrol..

[44] Aydin Z., Özcan Ş. (2026). Education and Decision Support Improve Outcomes in Urgent Dialysis: A Randomised Trial. Patient Educ. Couns..

[45] Naseri-Salahshour V., Sajadi M., Nikbakht-Nasrabadi A., Davodabady F., Fournier A. (2020). The Effect of Nutritional Education Program on Quality of Life and Serum Electrolytes Levels in Hemodialysis Patients: A Single-Blind Randomized Controlled Trial. Patient Educ. Couns..

[46] Hu Y., Zhang B., Hu Z., Huang J., Wang L., Wei Y., Zheng B., Xue Q. (2024). The Effect of Coaching on Health Information Literacy in Patients with Chronic Kidney Disease: A Randomized Controlled Trial. Trials.

[47] Bahmani M., Bijani M., Fereidouni Z., Dehghan A., Modreki A. (2025). Investigating the Impact of Interdisciplinary Training Programs on Self-Efficacy and Life Satisfaction among Hemodialysis Patients: A Randomized Controlled Clinical Trial. BMC Nephrol..

[48] Clark-Cutaia M.N., Sevick M.A., Thurheimer-Cacciotti J., Hoffman L.A., Snetselaar L., Burke L.E., Zickmund S.L. (2019). Perceived Barriers to Adherence to Hemodialysis Dietary Recommendations. Clin. Nurs. Res..

[49] Wong K.K., Velasquez A., Powe N.R., Tuot D.S. (2018). Association between Health Literacy and Self-Care Behaviors among Patients with Chronic Kidney Disease. BMC Nephrol..

[50] Mafra D., Leal V.O. (2016). A Practical Approach to a Low Protein Diet in Brazil. BMC Nephrol..

[51] Verseput C., Piccoli G. (2017). Eating Like a Rainbow: The Development of a Visual Aid for Nutritional Treatment of CKD Patients. A South African Project. Nutrients.

[52] Martins C.T.B., Biavo B.M.M., Uezima C.B.B., Santos J.A.P.D., Barros C.M.D., Ribeiro Júnior E., Troconis P.C., Scavone C., Luiz M.V.D.S.J. (2017). EPIC Trial: Education Programme Impact on Serum Phosphorous Control in CKD 5D Patients on Hemodialysis. J. Bras. Nefrol..

[53] Elder G.J., Malik A., Lambert K. (2018). Role of Dietary Phosphate Restriction in Chronic Kidney Disease. Nephrology.

[54] Kelly J.T., Warner M.M., Conley M., Reidlinger D.P., Hoffmann T., Craig J., Tong A., Reeves M., Johnson D.W., Palmer S. (2019). Feasibility and Acceptability of Telehealth Coaching to Promote Healthy Eating in Chronic Kidney Disease: A Mixed-Methods Process Evaluation. BMJ Open.

[55] Kelly J.T., Conley M., Hoffmann T., Craig J.C., Tong A., Reidlinger D.P., Reeves M.M., Howard K., Krishnasamy R., Kurtkoti J. (2020). A Coaching Program to Improve Dietary Intake of Patients with CKD: ENTICE-CKD. Clin. J. Am. Soc. Nephrol..

[56] Milazi M., Douglas C., Bonner A. (2018). A Bundled Phosphate Control Intervention (4Ds) for Adults with End-stage Kidney Disease Receiving Haemodialysis: A Cluster Randomized Controlled Trial Protocol. J. Adv. Nurs..

[57] Milazi M., Douglas C., Bonner A. (2021). A Bundled Phosphate Control Intervention (4Ds) for Adults with End-stage Kidney Disease Receiving Haemodialysis: A Cluster Randomized Controlled Trial. J. Adv. Nurs..

[58] Michail A., Andreou E. (2025). A Plant-Dominant Low-Protein Diet in Chronic Kidney Disease Management: A Narrative Review with Considerations for Cyprus. Nutrients.

[59] Liebman S.E., Baran A., Barnett T.D., Campbell T.M., Chen L., Friedman S.M., Hasan S., Le T.H., Monk R.D., Sabescumar J. (2025). The Effects of a Whole-Food Plant-Based Nutrition Education Program on Blood Pressure and Potassium in Chronic Kidney Disease: A Proof-of-Concept Study. Nutrients.

[60] Ekbote A., Ghosh-Jerath S., Sharma V., Subbaiyan S.S., Shah K.D., Joshi V.R., Ankush G.R., Sharma S., Kasiviswanathan S. (2024). Nutrition Profile and Quality of Life of Adult Chronic Kidney Disease Patients on Maintenance Hemodialysis in India: An Exploratory Study. Indian J. Nephrol..

[61] Costa N.A., Pereira A.G., Dorna M.D.S., Rodrigues H.C.N., Azevedo P.S., Paiva S.A.R., Polegato B.F., Balbi A.L., Zornoff L.A.M., Ponce D. (2021). Meal Timing and Frequency Implications in the Development and Prognosis of Chronic Kidney Disease. Nutrition.

[62] Di Lorenzo M., Lonardo M.S., Di Lauro M., Chiurazzi M., De Giovanni Di Santa Severina A.F., Capuano M., Guida B., Trio R., Pacella D., Memoli A. (2025). Real-World Snapshot of Dietary Patterns in Subjects Living with Chronic Kidney Disease. Nutrients.

[63] Sharma S., Alexander K.E., Green T., Wu M.-L., Bonner A. (2025). Energy Conservation Education Intervention for People with End-Stage Kidney Disease Receiving Haemodialysis (EVEREST): A Two-Arm Parallel Group Study. Int. J. Nurs. Stud..

[64] Christensen K.M., Bauer E.H., Prinds C. (2024). Exploration of Low-phosphate Diet Management of Patients Receiving Renal Dialysis: An Interpretive Description. J. Ren. Care.

[65] Somaili M., Akoor A., Refaei E., Deibaji M., Aqeel A.A., Ghulaysi S., Areeshi A.S., Mobaraki R.A., Qadah E.A., Gharwi N. (2025). Cross-Sectional Study for Assessment of Knowledge, Attitudes and Practices of Chronic Kidney Disease Patients toward Potassium-Rich Diet Intake in Jazan-Saudi Arabia. Medicine.

[66] Dsouza B., Prabhu R., Unnikrishnan B., Ballal S., Mundkur S.C., Chandra Sekaran V., Shetty A., Moreira P. (2023). Effect of Educational Intervention on Knowledge and Level of Adherence among Hemodialysis Patients: A Randomized Controlled Trial. Glob. Health.

[67] Sajjadi S.L., Ghafourifard M., Khosroshahi H.T. (2024). The Effect of Individualized Education on Learning Needs of Patients Undergoing Hemodialysis: A Randomized Controlled Clinical Trial. BMC Nephrol..

[68] Padial M., Taylor A., Sabatino A., Piccoli G.B., Avesani C.M. (2024). From Ultra-Processed Foods towards Healthy Eating for CKD Patients: A Proposal of Educational Infographics. J. Nephrol..

[69] Biruete A., Hill Gallant K.M., Lloyd L., Meade A., Moe S.M., St-Jules D.E., Kistler B.M. (2023). ‘Phos’Tering a Clear Message: The Evolution of Dietary Phosphorus Management in Chronic Kidney Disease. J. Ren. Nutr..

[70] Kim M., Koh J., Cho J., Cho S., Lee S., Huh H., Kim S., Jung S., Kang E., Park S. (2025). Association Between Healthy Dietary Patterns and Chronic Kidney Disease in Patients with Diabetes: Findings from Korean National Health and Nutrition Examination Survey 2019–2021. Nutrients.

[71] Martin T., Pringle L. (2020). Eating Out for Patients with Chronic Kidney Disease. J. Ren. Nutr..

[72] Taşkin Duman H., Karadakovan A. (2024). The Effect of Video Training on Symptom Burden, Comfort Level, and Quality of Life in Hemodialysis Patients: Clustered Randomized Controlled Trial. Patient Educ. Couns..

[73] Huang H.-L., Hsu Y.-H., Yang C.-W., Hsu M.-F., Chung Y.-C. (2024). Effects of a Health Literacy Education Program on Mental Health and Renal Function in Patients with Chronic Kidney Disease: A Randomized Controlled Trial. J. Nurs. Res..

[74] Morris A., Love H., Van Aar Z., Liles C., Roskell C. (2018). Integrating Renal Nutrition Guidelines into Daily Family Life: A Qualitative Exploration. J. Hum. Nutr. Diet..

[75] Gómez-García E.F., Cueto-Manzano A.M., Martínez-Ramírez H.R., Cortés-Sanabria L., Avesani C.M., Orozco-González C.N., Rojas-Campos E. (2024). Dietary Counseling, Meal Patterns, and Diet Quality in Patients with Type 2 Diabetes Mellitus with/without Chronic Kidney Disease. J. Diabetes Complicat..

[76] Joboshi H., Oka M. (2017). Effectiveness of an Educational Intervention (the Encourage Autonomous Self-Enrichment Program) in Patients with Chronic Kidney Disease: A Randomized Controlled Trial. Int. J. Nurs. Stud..

[77] Massey E.K., Gregoor P.J.H.S., Nette R.W., Van Den Dorpel M.A., Van Kooij A., Zietse R., Zuidema W.C., Timman R., Busschbach J.J., Weimar W. (2016). Early Home-Based Group Education to Support Informed Decision-Making among Patients with End-Stage Renal Disease: A Multi-Centre Randomized Controlled Trial. Nephrol. Dial. Transplant..

[78] McKinnon L., Giskes K., Turrell G. (2014). The Contribution of Three Components of Nutrition Knowledge to Socio-Economic Differences in Food Purchasing Choices. Public Health Nutr..

[79] Bradette-Laplante M., Carbonneau É., Provencher V., Bégin C., Robitaille J., Desroches S., Vohl M.-C., Corneau L., Lemieux S. (2017). Development and Validation of a Nutrition Knowledge Questionnaire for a Canadian Population. Public Health Nutr..

[80] Sandri E., Capoferri M., Piredda M., Micheluzzi V. (2025). Culinary Habits and Health: Analyzing the Impact of Cooking Practices and Knowledge Among Spanish Young Adults. Nutrients.

[81] Geaney F., Fitzgerald S., Harrington J.M., Kelly C., Greiner B.A., Perry I.J. (2015). Nutrition Knowledge, Diet Quality and Hypertension in a Working Population. Prev. Med. Rep..

[82] Vidgen H.A., Gallegos D. (2014). Defining Food Literacy and Its Components. Appetite.

[83] Short F. (2003). Domestic Cooking Practices and Cooking Skills: Findings from an English Study. Food Serv. Technol..

[84] Short F. (2003). Domestic Cooking Skills—What Are They?. J. HEIA.

[85] Kaesler N., Baid-Agrawal S., Grams S., Nadal J., Schmid M., Schneider M.P., Eckardt K.-U., Floege J., Bergmann M.M., Schlieper G. (2021). Low Adherence to CKD-Specific Dietary Recommendations Associates with Impaired Kidney Function, Dyslipidemia, and Inflammation. Eur. J. Clin. Nutr..

[86] Naber T., Purohit S. (2021). Chronic Kidney Disease: Role of Diet for a Reduction in the Severity of the Disease. Nutrients.

[87] Cupisti A., Gallieni M., Avesani C.M., D’Alessandro C., Carrero J.J., Piccoli G.B. (2020). Medical Nutritional Therapy for Patients with Chronic Kidney Disease Not on Dialysis: The Low Protein Diet as a Medication. J. Clin. Med..

[88] Lambert K., Bahceci S., Harrison H., Chan M., Scholes-Robertson N., Johnson D.W., Yip A., Viecelli A.K. (2022). The Caring for Australians and New Zealanders with Kidney Impairment (CARI) Guideline Group. Commentary on the 2020 Update of the KDOQI Clinical Practice Guideline for Nutrition in Chronic Kidney Disease. Nephrology.

[89] Mafra D., Brum I., Borges N.A., Leal V.O., Fouque D. (2025). Low-protein Diet for Chronic Kidney Disease: Evidence, Controversies, and Practical Guidelines. J. Intern. Med..

[90] Clark-Cutaia M.N., Ren D., Hoffman L.A., Snetselaar L., Sevick M.A. (2013). Psychometric Validation of the Self-Efficacy for Restricting Dietary Salt in Hemodialysis Scale. Top. Clin. Nutr..

[91] St-Jules D.E., Woolf K., Pompeii M.L., Kalantar-Zadeh K., Sevick M.A. (2016). Reexamining the Phosphorus–Protein Dilemma: Does Phosphorus Restriction Compromise Protein Status?. J. Ren. Nutr..

[92] McAuley E.A., Ross L.A., Hannan-Jones M.T., MacLaughlin H.L. (2025). Diet Quality, Self-Efficacy, and Health Literacy in Adults with Chronic Kidney Disease: A Cross-Sectional Study. J. Ren. Nutr..

[93] Talbot-Titley S.O., Mullan A.W.F., Lambert K. (2025). Development and Implementation of a Novel Approach to Dietary Education for People with Inadequate Health Literacy and Advanced Kidney Disease. J. Ren. Nutr..

[94] Ladsten T., Sarakatsannis Z. (2019). Your Guide to Create a Balanced Kidney-Friendly Meal. J. Ren. Nutr..

[95] Ferrara F., Siligato R., Di Maria A., Scichilone L., Di Simone E., Bondanelli M., Storari A., De Giorgi A., Di Muzio M., Fabbian F. (2023). Food Insecurity and Kidney Disease: A Systematic Review. Int. Urol. Nephrol..

[96] Crews D.C., Kuczmarski M.F., Grubbs V., Hedgeman E., Shahinian V.B., Evans M.K., Zonderman A.B., Burrows N.R., Williams D.E., Saran R. (2014). Effect of Food Insecurity on Chronic Kidney Disease in Lower-Income Americans. Am. J. Nephrol..

