# Supplementary material for: The Challenges of Establishing an Optimal Link Between Nutritional Requirements, Dietary Knowledge and Culinary Skills in Chronic Kidney Disease: An Exploratory Review of the Literature
Source: Nutrients. 2026 Jul 9;18(14):2245. doi: 10.3390/nu18142245 (PMC13415045; doi:10.3390/nu18142245)
Supplement: Supplementary file 1 [file nutrients-18-02245-s001.zip › nutrients-4395047-supplementary.pdf]

SECTION A: Maintenance Hemodialysis Cohorts

Authors	Study Design	Sample Size (N)	Clinical Characteristics (CKD)	Nutritional Needs	Culinary Knowledge	Clinical Correlations	Conclusions and Implications	Risk of Bias (JBI)
**[70]**	Randomized controlled trial	86 patients (43 DTSEP, 43 DEP)	End-stage renal disease starting dialysis under emergency conditions	The program includes reinforcement of adherence behaviors to diet and fluid management	Not specified	Structured education improved serum phosphorus and albumin levels, as well as general adherence	Decision support (Decision Tree) improves clinical and psychosocial outcomes in urgent dialysis.	Low
**[87]**	Randomized controlled trial	130 assigned; 105 completed (51 Psychoeducation, 54 CBT)	End-stage renal disease (ESRD) on chronic hemodialysis with depression	Education on healthy nutrition included in the third psychoeducation session	Not specified	Culinary skills were not analyzed; focused on reducing depressive symptoms through CBT vs. Psychoeducation	Psychoeducation was superior to CBT in reducing depression. Limited by a single-country sample.	Low
**[66]**	Non-blinded randomized controlled trial	100 patients (50 intervention, 50 control)	Hemodialysis patients in southern Iran	A dietitian was part of the interdisciplinary team educating on dietary adherence (salt and fluid reduction)	Not specified	The interdisciplinary program (including nutrition) significantly improved self-efficacy and life satisfaction	The interdisciplinary approach has transformative potential to improve psychological outcomes and quality of life.	Moderate (Lack of blinding)

**[68]**	Randomized clinical trial	102 patients (51 intervention, 51 control)	Hemodialysis patients	Personalized education based on individual learning needs and preferences.	Not specified	Individualized education (including diet and self-care) significantly reduced unmet learning needs.	Individualized education approach is more effective than traditional methods (brochures) for patient empowerment.	Low
**[79]**	Two-arm parallel study (pilot study)	50 participants (25 intervention, 25 control)	End-stage renal disease on hemodialysis with clinically relevant fatigue.	Not specified	Not specified	Not specified	The EVEREST program (energy conservation) is feasible and acceptable, showing potential reduction in fatigue. Larger-scale studies are required.	Low
**[52]**	Randomized controlled trial	100 patients	Hemodialysis patients	Educational intervention based on the empowerment model for diet and weight management.	Not specified	Not specified	The empowerment model significantly improved nutritional status and reduced fluid overload complications.	Low
**[53]**	Randomized controlled trial	112 patients (56 intervention, 56 control)	Maintenance hemodialysis patients	Education on therapeutic compliance, including diet and fluid restrictions using multimodal methods.	Not specified	Not specified	The combination of multimodal education and the "teach-back" method significantly improves self-management, health literacy, and quality of life.	Low

**[48]**	Randomized controlled trial	47 patients (23 intervention, 24 control)	Hemodialysis patients with PTH level monitoring	Standard nutritional education vs. education in specific food processing methods (renal cooking).	Intervention included teaching techniques to reduce phosphorus and calcium (specific boiling) and consuming foods prepared accordingly.	Practical culinary education together with a controlled diet significantly reduced PTH levels and maintained serum calcium.	Integrating food preparation techniques into dietary education improves mineral-bone metabolism control.	Low
**[71]**	Cluster Randomized Controlled Trial	48 patients (26 intervention, 22 control)	Maintenance hemodialysis patients	General education on symptom management, including diet and fluids, through informative videos.	Not specified	Not specified	Video training significantly reduced symptom burden and improved comfort level and quality of life.	Low
**[42]**	Qualitative study (Focus groups)	22 patients	Patients with ESRD on hemodialysis	Gastrointestinal symptoms and food taste directly influence dietary choices. There is a gap between knowledge and dietary behavior.	Patients report difficulties cooking food that meets restrictions while remaining palatable for their families.	Patients with better self-management and food preparation skills reported fewer symptoms of fluid overload.	Education should focus on realistic meal preparation strategies and symptom management to improve quality of life and adherence.	Low

**[67]**	Single-blind randomized clinical trial	74 patients (37 intervention, 37 control)	Maintenance hemodialysis patients	Nutritional education on sodium, potassium, phosphorus, and fluid intake through E-learning.	Not specified	Not specified	The multimedia educational program significantly improved quality of life and reduced phosphorus and potassium levels.	Low
**[62]**	Randomized clinical trial protocol	120 projected patients	End-stage renal disease on hemodialysis	Evaluation of a behavioral intervention package for phosphorus self-care.	Not specified (Protocol focused on behavioral education)	Not specified	Defines the methodology to evaluate if patient empowerment improves biochemical control.	Low (Rigorous design criteria)
**[63]**	Qualitative study (Interviews)	18 patients	Hemodialysis patients with adherence difficulties	Patients face the challenge of reconciling fluid, potassium, and phosphorus restrictions with cultural and social preferences.	Patients expressed not knowing how to prepare "renal" meals that taste good, leading to frustration and non-compliance.	Not specified	Lack of practical support and specific culinary skills contributes significantly to dietary non-adherence.	Low
**[60]**	Cluster Randomized Controlled Trial (Cluster RCT)	12 centers (463 patients)	End-stage renal disease (ESRD) on hemodialysis	Psychosocial intervention to improve adherence to fluid restriction and diet (potassium/phosphorus).	Not specified	Not specified	The SELF-HELP intervention significantly improved serum phosphorus control and interdialytic weight gain at 6 months.	Low

**[65]**	Cluster randomized controlled trial	120 patients (60 intervention, 60 control)	End-stage renal disease on hemodialysis with hyperphosphatemia	"4Ds" intervention (Diet, Drinks, Drugs, Dialysis) for phosphorus control.	Not specified	Not specified	The 4Ds intervention significantly improved knowledge but did not independently reduce serum phosphorus levels.	Low
**[64]**	Qualitative study (Semi-structured interviews)	35 patients	Maintenance hemodialysis patients.	Patients describe the renal diet as an overwhelming burden causing social isolation and confusion due to conflicting advice.	Patients express frustration at not knowing how to cook food that is simultaneously tasty, cultural, and kidney-"safe".	Inability to reconcile traditional culinary skills with renal restrictions is identified as a barrier to interdialytic weight control.	Education should move away from prohibitions and focus on practical support, empowerment, and cultural adaptation of cooking.	Low
**[72]**	Prospective intervention study (EPIC Trial)	63 patients	Stage 5D CKD on hemodialysis with hyperphosphatemia	Program focused on serum phosphorus control through the Transtheoretical Model of behavior change.	Not specified	Not specified	The stage-of-change-based educational program is effective in reducing serum phosphorus levels and improving nutritional adherence.	Low
**[49]**	Randomized controlled trial	47 patients (23 intervention, 24 control)	Maintenance hemodialysis patients	Education focused on phosphorus reduction through food selection and processing techniques.	Teaches specific thermal processing techniques (prolonged boiling in large water volumes)	Application of boiling culinary techniques significantly reduced serum phosphorus without	Practical culinary education is more effective than standard education for controlling hyperphosphatemia in hemodialysis patients.	Low

					to reduce phosphorus.	decreasing albumin (protein).		
**[54]**	Single-arm, non-randomized trial	366 patients	Hemodialysis patients with hyperphosphatemia	Intensive education program based on the "First Principles of Instruction" model for phosphorus control.	Includes demonstration and application of practical skills to identify hidden phosphorus and prepare suitable menus.	Increased practical skills through intensive education correlated with a significant and sustained reduction in serum phosphorus.	A structured and participatory educational program drastically improves phosphorus control compared to routine care.	Moderate (No randomized control group)
**[41]**	Cross-sectional descriptive study	38 patients	Patients in a hemodialysis program	Insufficient caloric intake was detected in 68% of the sample, and protein deficiencies in 47%.	Not specified	Not specified	There is a high risk of protein-energy malnutrition in hemodialysis patients, requiring constant nutritional monitoring.	Low

SECTION B: Non-Dialysis Dependent Advanced CKD Cohorts (Stages 3-5)								
**[57]**	Prospective cohort study	1,467 patients	Non-dialysis dependent CKD	Evaluation of diet quality using the Healthy Eating Index (HEI-2015).	Not specified	Higher diet quality was associated with a 43% reduction in the risk of CKD progression.	A healthy diet protects against disease progression and reduces the risk of all-cause mortality.	Low
**[85]**	Narrative review	94 references analyzed	Non-dialysis dependent CKD	Plant-dominant low-protein diet (PLADO). Focus on fiber and acid load reduction.	Discusses the use of herbs, spices, and techniques to improve the flavor of plant-based diets.	The use of culinary skills to substitute animal proteins with plant proteins improves the metabolic profile and reduces toxins.	The PLADO diet is promising for slowing CKD progression but requires personalization according to local culture.	Low
**[86]**	Cross-sectional study	309 patients	Non-dialysis dependent Chronic Kidney Disease (CKD) (stages 3-5)	75.1% of patients did not meet dietary fiber intake guidelines. High prevalence of non-compliance with potassium and sodium recommendations.	Not specified	Not specified	Low nutritional health literacy is independently associated with lower fiber intake. Communication skills of health professionals need to be improved.	Low
**[55]**	Cross-sectional observational study	126 patients	CKD stages 3 to 5	Assessment of salt intake through 24-hour urinary sodium excretion.	Not specified	No significant correlation was found between salt knowledge and actual sodium intake.	Theoretical knowledge alone is not sufficient to reduce salt consumption; behavioral changes are required.	Low

**[56]**	Observational study	155 patients	CKD (stages 3-5)	Direct correlation exists between high salt intake and the progression of proteinuria and hypertension.	Not specified	Not specified	Regular monitoring of sodium intake is essential to prevent deterioration of renal function.	Low
**[38]**	Proof-of-concept study	18 patients	CKD (stages 3-4)	Whole Food Plant-Based (WFPB) nutrition education program to evaluate safety and potassium levels.	The program included practical culinary demonstrations for preparing low-sodium plant-based recipes.	Better culinary skills for preparing vegetables allowed for a safe transition to plant-based diets without hyperkalemia.	A WFPB diet is feasible and safe in CKD; it significantly reduced systolic blood pressure without elevating potassium levels.	Moderate (Small sample and short duration)
**[69]**	Cross-sectional observational study	404 patients	CKD at various stages in Jazan, Saudi Arabia.	Assessment of knowledge regarding potassium-rich diet; 53.5% of patients had deficient knowledge.	55.7% of patients use techniques such as soaking vegetables to reduce potassium content.	Significant correlation exists between educational level and potassium knowledge, affecting dietary practices.	Crucial to implement specific nutritional education programs to improve potassium management and prevent hyperkalemia.	Low
**[40]**	Cross-sectional study (KARE trial analysis)	137 patients	Low-income patients with CKD (stages 3 and 4)	Evaluation of the association between health literacy and behaviors such as fast food and sugary drink consumption.	Not specified	Lower health literacy correlated with significantly lower fast food consumption (possibly due to lower access or cost).	Health literacy influences self-care behaviors, but socioeconomic barriers also mediate dietary choices.	Low

**[89]**	Qualitative study	27 patients	CKD (stages 3-5)	Patients demand information on how to eat healthily instead of only receiving lists of forbidden foods.	Patients identify the lack of skills to cook tasty, low-salt dishes as the main barrier to adherence.	Not specified	A paradigm shift is necessary toward educational programs that teach practical culinary skills instead of just theory.	Low
**[18]**	Qualitative study (Semi-structured interviews)	21 patients	Advanced Chronic Kidney Disease (stages 4 and 5) under conservative treatment (non-dialysis)	Patients perceive the renal diet as highly restrictive, causing confusion and social isolation. They prefer instructions on what to eat rather than what to avoid.	Lack of skills to adapt traditional recipes to renal restrictions was identified as a major barrier to adherence.	Not specified	Adherence is facilitated by family support and practical knowledge but hindered by the loss of pleasure in eating and information complexity.	Low
**[61]**	Cross-sectional study (survey)	133 patients	CKD (stages 3-5)	A significant gap was identified between theoretical knowledge of guidelines and the ability to apply them at the table.	Most patients report medium confidence in the kitchen but low ability to substitute forbidden ingredients with healthy alternatives.	Not specified	Improving confidence in culinary skills is a key therapeutic goal to enhance the nutritional status of renal patients.	Low

**[28]**	Innovation program / Educational intervention	>400 Veterans	CKD stage 3	Protein restriction (0.6-0.8 g/kg) and sodium to slow disease progression.	Includes the "Healthy Teaching Kitchen," where live culinary demonstrations prepare renal recipes.	Practical learning in the "Teaching Kitchen" improves patient confidence to apply theoretical restrictions.	Early intervention with practical culinary components closes knowledge gaps and motivates lifestyle changes.	Low
**[58]**	Randomized controlled trial	112 patients (67 intervention, 45 control)	Pre-terminal CKD (Pre-ESRD) patients	Self-care intervention for diet management, blood pressure control, and treatment adherence.	Not specified	Not specified	The self-care program significantly improved renal function (eGFR), reduced proteinuria, and decreased depression levels.	Low
**[95]**	Patient education article (Clinical guide)	16 final references analyzed	CKD (non-dialysis dependent)	Practical guide on protein restriction (0.6-0.8 g/kg), phosphorus, potassium, and sodium control.	Provides culinary techniques such as leaching and boiling of tubers to reduce potassium.	Not specified	Structured meal planning and the use of specific cooking techniques are key to delaying disease progression.	Low

**[74]**	Practical approach review (Correspondence)	33 final references analyzed	CKD stages 3-5 (non-dialysis) in the context of Brazil	Low-protein diet (LPD: 0.6 g/kg) adapted to Brazilian habits of high red meat and legume consumption.	Recommends the use of practical recipes ("Low Protein & High Flavor") to facilitate the transition to a tasty hypoproteic diet.	Intensive and specialized nutritional counseling allows adapting traditional dishes (rice and beans) to maintain adherence.	Successful implementation of LPD requires considering local culinary culture and overcoming economic barriers of the health system.	Low
**[13]**	Qualitative study (Interviews and focus groups)	49 patients	CKD across the spectrum (pre-dialysis, hemodialysis, peritoneal dialysis, and transplant)	Patients identify healthy food costs and lack of variety as critical barriers to dietary adherence.	Lack of skills to prepare renal meals that are tasty and culturally acceptable is highlighted as a primary barrier.	Not specified	Adherence requires an approach combining social support, practical education, and financial barrier reduction.	Low

SECTION C: Translational Clinical Guides and Methodological Frameworks								
**[73]**	Systematic review	22 studies	Chronic Kidney Disease (pre-dialysis and dialysis)	Educational interventions significantly reduce serum phosphorus levels. The use of visual tools and motivational interviews improves outcomes.	Not specified	Not specified	Nutritional education is fundamental for metabolic control in CKD. No single educational method was found superior to others.	Low
**[81]**	Systematic review	24 studies	Chronic Kidney Disease (CKD)	Educational interventions significantly improve diet knowledge and serum phosphorus control.	Not specified	Not specified	Educational support is vital for self-management, although greater methodological rigor is required in future studies.	Low
**[39]**	Narrative review / Review article	47 final references analyzed	CKD at various stages (emphasis on CKD-MBD and phosphorus)	Prioritize plant proteins (lower bioaccessibility), avoid phosphorus additives, and optimize the phosphorus-protein ratio	Discusses the impact of methods such as soaking, fermentation, and germination to reduce phytates and phosphorus	Suggests that the use of specific culinary techniques can reduce phosphorus load without compromising protein intake	Phosphorus management has evolved from numerical targets to an approach based on sources and bioaccessibility.	Low (Comprehensive review of current evidence)

**[88]**	Point of view / Expert opinion article	Not specified (Tool proposal)	Chronic Kidney Disease (CKD)	Reduction of ultra-processed foods due to high content of added sodium, phosphates, and potassium.	Proposes the use of infographics to transform ultra-processed foods into "Healthy Eating" home-cooked meals.	Not specified	Visual tools (infographics) facilitate the substitution of industrial products with natural and safe culinary options.	Low
**[75]**	Concept paper / Visual tool development	Not specified (Context review)	CKD in South African context (ethnic diversity and poverty)	Need for personalized diets considering lack of refrigeration and the habit of sharing "from the pot."	Introduces the "Eating Like a Rainbow" visual aid to teach cooking techniques that reduce sodium and improve protein quality.	Empowerment through visual culinary education seeks to mitigate malnutrition and sodium excess in monotonous diets.	Culturally adapted visual tools are essential to overcome literacy and resource barriers in renal management.	Low
**[76]**	Descriptive review / Practical approach	23 final articles cited	CKD (pre-dialysis and dialysis) in poverty contexts in South Africa	Protein restriction (0.6-0.8 g/kg in pre-dialysis), strict control of sodium (80-100 mmol/day) and potassium	Use of the "South African Renal Exchange List" and hand measurements (fist/palm) to manage portions	Empowerment in home food preparation allows for greater control over ingredients and kidney health	Nutritional success depends on adapting global guidelines to local racial, cultural, and socioeconomic realities.	Low (Based on established clinical protocols)

**[47]**	Narrative review	75 final articles analyzed	Chronic Kidney Disease (CKD), including hemodialysis patients	Focus on low-protein (LPD) and very-low-protein (vLPD) diets to delay progression and control uremic toxins.	Discusses the role of "culinary skills" in improving the palatability of low-sodium and low-protein foods, facilitating long-term adherence.	Better culinary skills are mentioned to allow salt substitution with spices, indirectly improving blood pressure control and uremic symptoms.	Diet must be personalized, and nutritional counseling is key to preventing malnutrition and improving renal prognosis.	Low
**[96]**	Patient education tool (Clinical guide)	4 final references analyzed	CKD (non-specific, applicable to pre-dialysis)	Practical guide for eating out: portion control, sodium, phosphorus, potassium, and menu selection.	Offers practical tips on culinary techniques in restaurants (ordering sauces on the side, avoiding salt shakers, substituting sides).	Not specified	Self-management through knowledge of portions and food selection techniques improves adherence outside the home.	Low
**[83]**	Multicenter randomized controlled trial	80 patients	End-Stage Renal Disease (ESRD) in the treatment decision phase	Group home education on renal replacement therapy options (dialysis vs. transplant).	Not specified	Not specified	Home education significantly increases knowledge and communication regarding treatment options, including living donor transplantation.	Low

**[3]**	Experimental study (Bromatological analysis)	10 types of food (Vegetables and meats)	Applicable to dialysis patients (nutrient analysis)	Analysis of how cooking affects proteins, calcium, and phosphorus to optimize hospital menus.	Evaluates methods: boiling, steaming, stewing, and roasting. Boiling reduces phosphorus by 27-46%, but hard water may increase calcium.	Use of boiling culinary techniques allows phosphorus load reduction without significant protein loss.	Preparation method is as important as food selection for controlling hyperphosphatemia.	Low
**[43]**	Qualitative study (Interviews and focus groups)	41 participants (patients, caregivers, and professionals)	Advanced stage CKD	Need for practical information on salt substitutes and managing restrictions without losing the pleasure of eating.	Patients demand cooking workshops and practical demonstrations to translate theoretical guidelines into real meals.	Not specified	It is crucial to move from prohibitive education ("what not to eat") to facilitator-based education using practical skills and cooking.	Low
**[93]**	Review article / Clinical perspective	27 final references analyzed	Hemodialysis patients with hyperphosphatemia.	Proposes reducing phosphorus without compromising protein by avoiding additives and selecting low-bioavailability sources.	Highlights the use of "moist cooking methods" (boiling) to leach phosphorus from food without losing protein quality.	Use of appropriate culinary techniques allows up to a 50% phosphorus reduction in certain foods, improving serum control.	Clinically relevant phosphorus restrictions are possible without compromising protein status through education in cooking techniques.	Low

**[45]**	Mixed-methods study (Digital intervention)	114 participants	CKD (stages 1-4)	Evaluation of the "My Kidneys & Me" platform providing education on self-management and renal diet.	The platform includes specific sections on meal preparation, recipes, and practical cooking tips.	Access to digital culinary resources improved patients' confidence in following dietary recommendations.	Digital interventions are usable and accepted tools that improve empowerment and dietary self-management.	Low
